# Dynamic variations in the ultrasound greyscale median of carotid artery plaques

**DOI:** 10.1186/1476-7120-11-21

**Published:** 2013-06-14

**Authors:** Baris Kanber, Timothy C Hartshorne, Mark A Horsfield, Andrew R Naylor, Thompson G Robinson, Kumar V Ramnarine

**Affiliations:** 1 Department of Cardiovascular Sciences, University of Leicester, Leicester, UK; 2 Department of Surgery, University Hospitals of Leicester NHS Trust, Leicester, UK; 3 NIHR Biomedical Research Unit for Cardiovascular Sciences, University of Leicester, Leicester, UK; 4 Department of Medical Physics, University Hospitals of Leicester NHS Trust, Sandringham Building, Leicester Royal Infirmary, Infirmary Square, Leicester LE1 5WW, UK

**Keywords:** Ultrasound, Carotid plaque, GSM, Frame-by-frame, Inter-frame, Variations

## Abstract

**Background:**

Several studies have found that the ultrasound greyscale median (GSM) of carotid artery plaques may be useful for predicting the risk of cerebrovascular events. However, measurements of GSM are typically performed on still ultrasound images ignoring any variations that may be observed on a frame-by-frame basis. The aim of this study was to establish the existence and investigate the nature and extent of these variations.

**Methods:**

Employing a novel method that enabled plaque boundaries to be tracked semi-automatically, variations in the plaque GSM and observed cross-sectional area were measured for 27 carotid artery plaques (19 consecutive patients, stenosis range 10%-80%) over image sequences of up to 10 seconds in length acquired with a mean frame rate of 32 frames per second.

**Results:**

Our results showed a mean inter-frame coefficient of variation (CV) of 5.2% (s.d. 2.5%) for GSM and 4.2% (s.d. 2.9%) for the plaque area. Thirteen of the 27 plaques (48%) exhibited CV in GSM greater than 5% whereas only six plaques (22%) had CV in plaque area of greater than 5%. There was no significant correlation between the CV of GSM and plaque area.

**Conclusions:**

Inter-frame variations in the plaque GSM such as those found in this study have implications on the reproducibility of GSM measurements and their clinical utility. Studies assessing the GSM of carotid artery plaques should consider these variations.

## Background

The North American Symptomatic Carotid Endarterectomy Trial (NASCET) and the European Carotid Surgery Trial (ECST) have shown that surgery in symptomatic patients with severe internal carotid artery stenosis results in a six-fold reduction of stroke risk [1,2]. However, patients who do not have severe stenoses and patients who are asymptomatic can also go on to develop stroke. It is, therefore, important to be able to determine whether any of these patients have carotid plaques which are high-risk or unstable. Ultrasound greyscale median (GSM) is commonly used to quantify the ultrasound appearance of carotid plaques, and several studies have found that it may be valuable for predicting the risk of cerebrovascular events. In particular, statistically significant associations have been reported between plaque GSM and the presence of cerebrovascular symptoms [3,4], cerebral infarction in symptomatic and asymptomatic patients [5-7], recurrent cerebrovascular events before undergoing carotid endarterectomy [8], and the overall risk of stroke in symptomatic patients [9], asymptomatic patients [10], and during and after carotid artery stenting [11].

GSM measurements currently have poor reproducibility across studies. This can be partly attributed to the differences in the acquisition settings used in separate studies. In order to reduce this variability, investigators have attempted to standardise ultrasonic images of carotid plaques by specifying certain acquisition settings to be used for carotid artery scanning and normalizing the resultant ultrasound images [12]. However, existing studies typically measured GSM on still ultrasound images, and thus ignored any variations that may have been observed on a frame-by-frame basis. The purpose of this study was to establish the existence of and investigate the nature and extent of any frame-by-frame variations in the plaque GSM using a novel technique for tracking plaque boundaries in ultrasound image sequences. We hypothesized that variations in the GSM of carotid artery plaques may occur due to deformation of the plaque during the cardiac cycle, and other confounding factors such as out-of-plane plaque, patient or probe motion. Furthermore, it was hypothesized that plaques of different composition and morphology may exhibit different inter-frame variations in GSM in otherwise equivalent hemodynamic circumstances and hence the measurement of these variations may give useful insight into the dynamic behaviour of plaques and help identify vulnerable plaques.

## Methods

### Data acquisition

Frame-by-frame variations in the plaque GSM and area of 27 carotid artery plaques (19 consecutive patients, 11 males, mean age 76, stenosis range 10%-80%) were studied by measuring the GSM and area of plaques on each image frame separately and computing the mean, the standard deviation (s.d.) and the coefficient of variation (s.d./mean) across the frames. The image sequences used were of up to 10 seconds in length (average 4.4 seconds) and were acquired with a mean frame rate of 32 frames per second. The degrees of stenosis of the corresponding arteries were measured using criteria consistent with the NASCET methodology utilizing blood flow velocities in conjunction with the B-Mode and colour flow imaging [2,13,14]. Eleven of the plaques studied were found to be asymptomatic and the remaining sixteen symptomatic after assessment at the University Hospitals of Leicester NHS Trust’s Rapid Access Transient Ischemic Attack (TIA) Clinic. The use of the clinical data for our research had been approved by the National Research Ethics Service (NRES) Committee East Midlands - Northampton (reference 11/EM/0249), and each patient gave informed consent before participating in the study. The ultrasound data were obtained as longitudinal cross-sections using a Philips iU22 ultrasound scanner (Philips Healthcare, Eindhoven, The Netherlands) with an L9-3 probe and included B-Mode (i.e. greyscale) and Colour Doppler image sequences. The vascular carotid preset on the scanner was used (Vasc Car preset, persistence low, XRES and SONOCT on) and the gain was optimized by the operator (TCH) who is an experienced vascular sonographer. In the case of B-Mode acquisitions, the greyscale transfer curve was kept set to Gray Map 2, as this was reported to be the most linear transfer curve on this scanner [15]. Colour Doppler cine-loops were used as a qualitative aid to identifying the location and extent of the plaques, while the B-Mode data were used for the quantitative analyses of the plaque GSM and cross-sectional area.

### Data analysis

Quantitative analyses were carried out using MATLAB version 7.14, release 2012a (MathWorks, Natick, Massachusetts, USA) and employed a combination of standard speckle tracking techniques and a novel surface tracking algorithm. The latter was used to delineate and track plaque-arterial lumen boundaries and was based on a probabilistic approach to vessel lumen segmentation [16]. In this approach, given a point B with probability P_b_ of being in the arterial lumen of interest, the probability P_a_ that a neighbouring point A was also part of the same lumen was proportional to P_b_ with a Gaussian fall in probability with increasing greyscale contrast between the two points (Equation 1). Here G_b_ and G_a_ were the greyscale intensities of points B and A, and the constant ζ was determined by considering the amount of greyscale contrast (G_th_) required to reduce P_a_ to 1/2 that of P_b_.

(1) $$\begin{array}{l} P_{a}=P_{b}\;\exp\left(-{\left(G_{b}-G_{a}\right)}^{2}/\zeta \right)\;\mathrm{where}\;\zeta \\ \;=G_{\mathrm{th}}{}^{2}/\log\;2 \end{array}$$

The arterial lumen tracking technique based on this algorithm was previously found to have good arterial wall tracking performance, comparable to that of Tissue Doppler Imaging [17].

Speckle tracking, which was used to track the plaque-underlying tissue boundaries, is a standard image analysis technique that involves measuring the similarity between a template and a search image [18]. Given a point to speckle track, a region is defined around the point and used as a template. The process is then essentially to find the position in the search image that has the largest similarity to this template. There are many different measures of similarity between a template and a search image; in this study the normalized correlation coefficient was used since it is invariant to changes in image amplitude [18]. Square regions of approximate area 1.4 × 1.4 cm^2^ were employed. This template size was found to produce optimum speckle tracking quality in our study as was verified by observing the plaque tracking results (Additional file 1).

**Figure 1:**
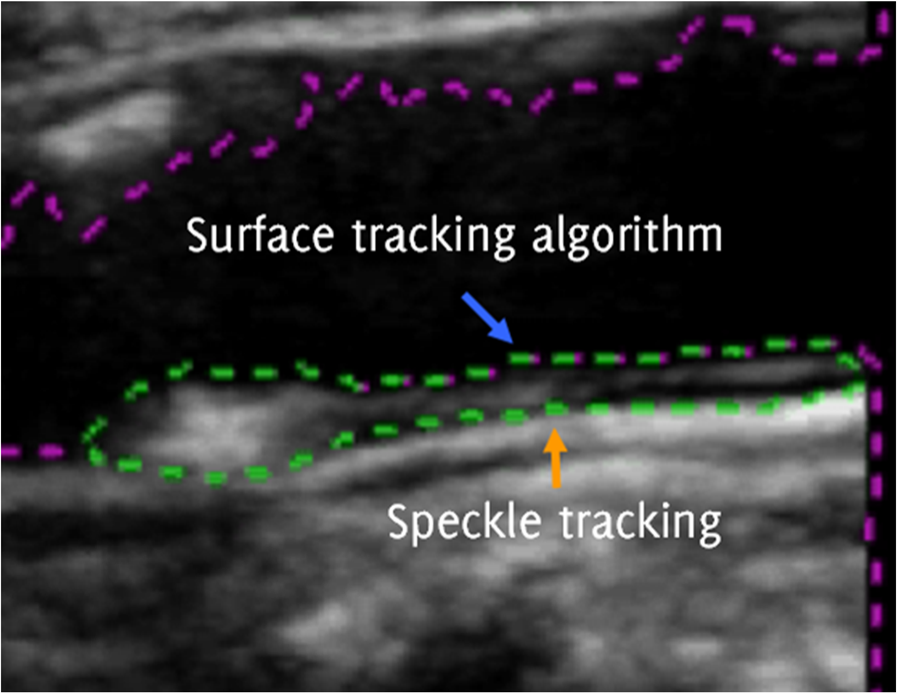
**The plaque region shown by the dashed green lines is defined by the two boundaries: the top boundary (blue arrow) defines the plaque-arterial lumen interface and the bottom boundary (orange arrow) defines the plaque-arterial wall boundary.** The purple lines are the output of the surface tracking algorithm.

Speckle tracking requires stable speckle patterns to be useful. Speckle patterns at plaque-arterial wall boundaries usually fulfil this requirement but speckle patterns at plaque-arterial lumen boundaries tend to de-correlate rapidly. For this reason, the arterial lumen segmented out using the surface tracking algorithm defining the plaque-arterial lumen boundary, was automatically cut and joined with a polygon comprising the speckle tracked points defining the plaque-arterial wall interface (Figure 1). The joining process was carried out by finding the closest points on the arterial lumen surface to the proximal and distal ends of the speckle-tracked plaque-arterial wall boundary and joining these respective points.

Regions of plaques that could not be distinguished from the arterial lumen (e.g. echo-free regions) and regions of plaques in areas of acoustic shadowing were excluded from analysis. Plaques for which anechoic regions and regions of shadowing exceeded more than 70% of the total plaque cross-sectional area as observed on Colour Doppler sequences were not included in the study.

Image normalisation was carried out using two different methods in order to observe their effects on the frame-by-frame variations observed. The first normalisation (NORM1) was performed by linearly scaling the ultrasound image intensities so that the GSM of a user-selected blood region inside the vessel lumen was mapped to 0 and the brightest region of the adventitia was mapped to 190. Both of these regions were 5 × 5 pixels in size, corresponding to an approximate area of 0.4 × 0.4 mm^2^. The reference regions were selected on the first image of the sequence and the reference GSM values calculated on the first frame were applied to that and all subsequent images. The second normalisation (NORM2) involved selecting blood and adventitia regions on each image separately, thus applying separate reference values to individual images.

A semi-qualitative assessment of whether physiologically reasonable (e.g. of the order of 60/min) periodical variations were visually apparent on the GSM and plaque area waveforms was also carried out. This involved measuring the frequency of any periodical variations seen on the GSM and cross-sectional area waveforms and considering frequencies in the range 50/min - 160/min to be potentially attributable to cardiac variations. Conversely, variations with frequencies lower than 50/min or higher than 160/min were not considered to be due to physiological sources and such plaques were placed in the same category as those not showing any apparent periodical variations in the GSM and cross-sectional area.

### Statistical methods

Statistical analyses were carried out using MATLAB version 7.14, release 2012a (MathWorks, Natick, Massachusetts, USA) and SPSS version 20 (IBM Corporation, Armonk, New York, USA). Spearman’s test was used to study the correlation between the inter-frame variations in GSM and area, since neither of these parameters was expected to follow a Gaussian distribution and any correlation between the two was likely to be non-linear. Multi-variable linear regression was used to study the contribution of other plaque GSM and area parameters to the differences observed in the magnitude of the GSM variations for each plaque. An unpaired, non-parametric Mann–Whitney U-test was used to investigate whether the GSM values averaged across all frames, as well as their standard deviations and the coefficients of variation, differed significantly between the asymptomatic and symptomatic plaque groups. Two-tailed values of significance were used in each case.

### Reproducibility

Intra-observer coefficients of variation for eight selected plaque samples of varying echogenicities were studied by measuring the frame-by-frame variations in the plaque GSM and cross-sectional area five times for each plaque. The measurements were made by the same operator (BK) and in sequential order. The same ultrasound acquisition sequences were used for each plaque respectively. The eight plaques concerned were selected from the available dataset to give a wide spectrum of plaque echogenicities for reproducibility analysis.

### Comparison against manual measurements

In order to compare the plaque GSM and cross-sectional areas obtained using our method with those obtained using manual delineation, plaque GSM and cross-sectional area were measured by the same operator (BK) using manual delineation for every 5th frame, for each of the same eight plaque samples used for our study of reproducibility. This enabled the magnitude of and variation in the plaque GSM and cross-sectional areas to be compared between the two techniques. A Bland-Altman analysis was also carried out to assess the agreement between the GSM measurements made using our method and manual delineation on matching image frames.

## Results

**Figure 2:**
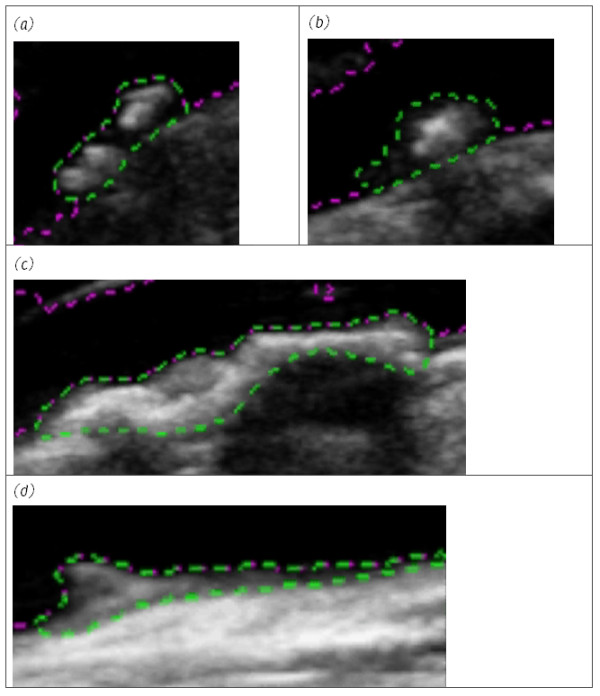
**Close-up views of four plaque samples with varying echogenicities (single frames shown).** Plaques (**a**) px1, (**b**) px3, (**c**) px19, (**d**) px22. The region of acoustic shadowing has been excluded from analysis for px19.

Plaque outlines could be tracked successfully in a variety of different configurations (Figure 2). Across all plaque samples, the un-normalized plaque GSM, averaged across all frames, ranged between 26 and 112 (mean 47, Table 1). Plaque areas ranged between 7 mm^2^ and 92 mm^2^ (mean 30 mm^2^). The mean inter-frame coefficient of variation (s.d./mean) of GSM was 5.2% (s.d. 2.5%) while that of plaque area was 4.2% (s.d. 2.9%). In relation to the normalized GSM obtained using the NORM1 method, the corresponding mean GSM figures ranged between 24 and 96 (mean 46). The mean inter-frame coefficient of variation was the same as without normalization (5.2%) but the standard deviation was slightly larger (2.6%). Normalization using the second normalisation technique (NORM2) for plaques px1, px2, px3, px19 (excluding the region of acoustic shadowing) and px22 resulted in larger coefficients of variation (4.8%, 9.7%, 6.7%, 3.8% and 4.9% respectively) compared to the un-normalized and NORM1 normalized coefficients of variation (Table 1).

**Table 1:** Variations observed in the plaque GSM and area

**Plaque sample**	**GSM (un-normalized)**			**GSM (normalized)**			**Area**			**Periodical variations observed?**
	**mean**	**s.d.**	**CV (s.d./mean)**	**mean**	**s.d.**	**CV (s.d./mean)**	**mean (mm**^**2**^**)**	**s.d. (mm**^**2**^**)**	**CV (s.d./mean)**	
px1	40.3	1.69	4.2%	40.5	1.69	4.2%	22.3	0.25	1.1%	GSM
px2	32.3	2.80	8.7%	27.5	2.38	8.7%	27.9	1.12	4.0%	
px3	25.6	1.52	5.9%	24.4	1.45	5.9%	16.9	0.69	4.1%	Both
px4	76.9	2.22	2.9%	71.0	2.08	2.9%	11.3	1.05	9.3%	
px5	36.9	4.31	11.7%	30.9	3.60	11.7%	22.0	0.96	4.4%	Both
px6	33.8	2.87	8.5%	36.1	3.06	8.5%	28.9	3.56	12.3%	Both
px7	33.6	0.84	2.5%	35.8	0.90	2.5%	52.5	1.14	2.2%	Both
px8	28.3	2.02	7.1%	32.9	2.53	7.7%	7.2	0.44	6.0%	Both
px9	29.6	1.19	4.0%	27.8	1.12	4.0%	21.9	0.59	2.7%	
px10	27.6	0.74	2.7%	26.3	0.70	2.7%	13.1	0.80	6.1%	
px11	70.2	1.27	1.8%	71.7	1.30	1.8%	38.0	0.23	0.61%	Area
px12	54.0	3.72	6.9%	61.0	4.28	7.0%	14.9	0.39	2.6%	
px13	39.3	3.12	7.9%	33.5	3.05	9.1%	14.6	1.65	11.3%	GSM
px14	40.9	1.66	4.1%	38.1	1.55	4.1%	30.2	1.29	4.3%	
px15	53.7	1.97	3.7%	53.1	1.95	3.7%	15.9	0.71	4.4%	
px16	31.9	1.68	5.3%	34.8	1.83	5.3%	14.6	0.93	6.4%	Area
px18	73.3	5.30	7.2%	58.3	4.21	7.2%	25.8	0.82	3.2%	
px19	112.3	1.98	1.8%	95.7	1.72	1.8%	67.0	1.14	1.7%	
px21	52.4	2.35	4.5%	51.1	2.34	4.6%	22.7	1.09	4.8%	GSM
px22	67.4	2.15	3.2%	56.7	1.80	3.2%	21.5	0.80	3.7%	Both
px23	30.1	0.64	2.1%	25.6	0.54	2.1%	49.4	1.22	2.5%	Excluded
px24	35.5	2.52	7.1%	36.9	2.62	7.1%	14.4	0.37	2.6%	Excluded
px25	82.9	6.02	7.3%	82.3	5.98	7.3%	37.2	0.61	1.6%	Excluded
px26	43.4	1.49	3.4%	55.3	1.90	3.4%	39.5	0.94	2.4%	
px27	50.1	1.17	2.3%	56.3	1.31	2.3%	66.1	1.68	2.5%	
px28	31.2	1.90	6.1%	42.4	2.58	6.1%	13.6	0.45	3.3%	Area
px29	33.9	2.20	6.5%	27.2	1.77	6.5%	91.9	1.93	2.1%	

The last column indicates whether periodical variations of the order of 60/min were seen on the inter-frame *GSM* and area waveforms. Normalized *GSM* refers to *NORM1*.

Periodic variations with frequencies of the order of 60/min in either or both of the plaque GSM or area waveforms were observed for 12 plaques (50%) but not observed for 12 other plaques (50%, Table 1). Three plaques were excluded from this analysis as they had short acquisition sequences.

**Figure 3:**
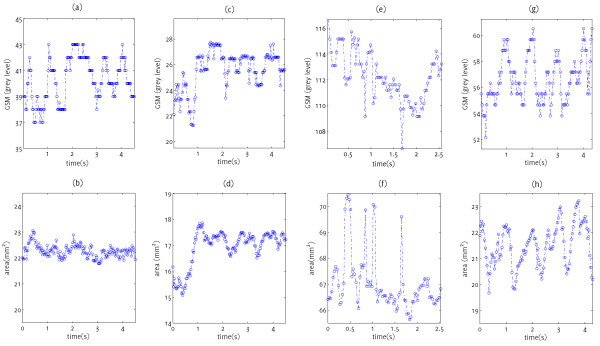
Variations in the un-normalized plaque GSM (top row), and plaque area (bottom row) for the plaque samples px1 (a,b), px3 (c,d), px19 (e,f), px22 (g,h).

Overall, 13 of the 27 plaques (48%) exhibited inter-frame variations in GSM of greater than 5% measured as the inter-frame coefficient of variation of GSM in both the un-normalized and NORM1 normalized cases. In contrast, only 6 of the 27 plaques (22%) had inter-frame coefficients of variation in plaque area of greater than 5% (Table 1, Figure 3).

**Figure 4:**
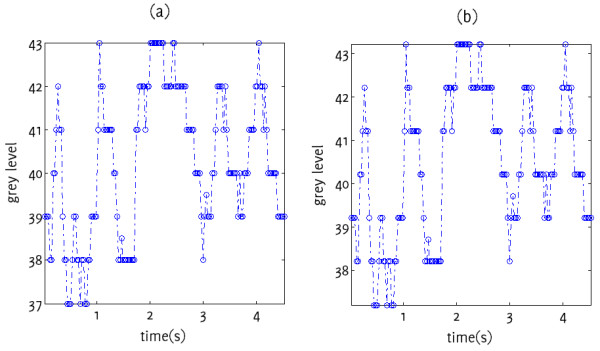
Variations in GSM for plaque sample px1: (a) un-normalized, (b) normalized (NORM1).

**Figure 5:**
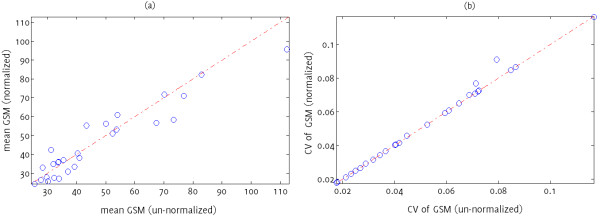
**Comparison of normalized and un-normalized plaque GSMs:** (**a**) NORM1 normalized mean GSM versus un-normalized, (**b**) NORM1 normalized coefficients of variation versus un-normalized. Red dashed lines indicate no change upon normalization.

Normalisation using NORM1 did not appear to change the shape of the GSM variation waveform but caused a translation along the y-axis (Figure 4). After normalization, the mean GSM was lower for some plaques, and higher for others (Figure 5a). The coefficients of variation were predominantly the same (Figure 5b), yet for some plaques, NORM1 also changed the coefficient of variation (Figure 5b).

**Figure 6:**
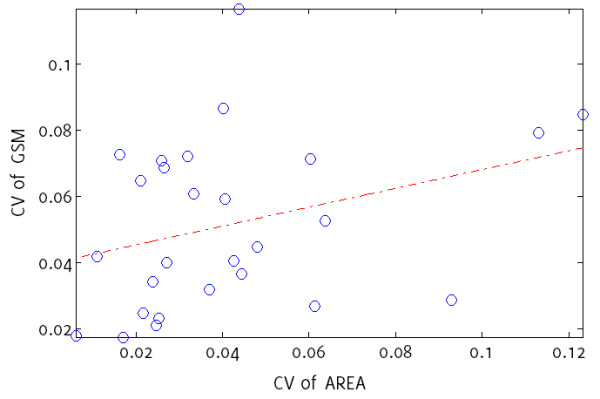
**Scatter plot of inter-frame coefficients of variation for un-normalized GSM versus those for plaque area.** The correlation between the two coefficients of variation is weak (Spearman’s rho 0.36, p = 0.07). The dashed line is a linear fit to the data.

The correlation between the inter-frame coefficients of variation in un-normalized plaque GSM and cross-sectional area (Figure 6) was not statistically significant (Spearman’s rho 0.36, p = 0.07). Testing the influences on the extent of the inter-frame variations seen in the un-normalized GSM, of (a) the mean inter-frame, un-normalized GSM, (b) the mean inter-frame plaque area, and (c) the extent of inter-frame variations seen in plaque area, with the extents taken as the standard deviation of inter-frame values, identified the mean inter-frame GSM as the only statistically significant factor at the 5% significance level (Table 2).

**Table 2:** Results of multi-variable linear regression, testing for the influences of (a) mean frame-by-frame GSM values, (b) mean frame-by-frame plaque areas, and (c) the standard deviations of the frame-by-frame plaque areas on the standard deviations of the frame-by-frame GSM values

**Factor**	**(a)**	**(b)**	**(c)**
**Standardized coefficient (β)**	0.48	−0.33	0.19
**t statistic**	2.46	−1.59	0.93
**Significance (p)**	0.02	0.13	0.36

**Figure 7:**
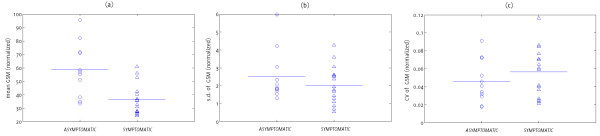
**Distribution of the (a) mean, normalized GSM, (b) standard deviations of the inter-frame GSM values, and (c) the coefficients of variation, for the symptomatic and asymptomatic plaque groups.** The horizontal lines indicate mean values for the individual groups.

The mean, normalized GSM differed significantly between the symptomatic and asymptomatic groups (p = 0.002) but the parameters based on the inter-frame variations in the normalized GSM did not (p = 0.48 for the inter-frame standard deviation of normalized GSM and p = 0.42 for the coefficient of variation of normalized GSM, Figure 7).

**Figure 8:**
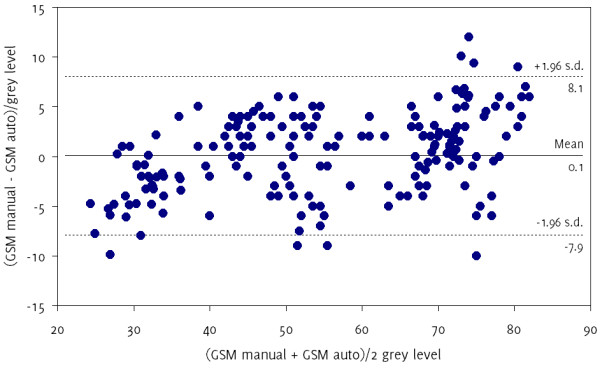
Bland-Altman plot showing the differences in GSM measurements on matching image frames between our method and manual delineation.

Our plaque GSM and area measurements showed good reproducibility (Table 3) and were broadly comparable to those obtained using manual delineation (Table 4). The mean intra-observer coefficients of variation for the eight selected plaque samples were 1.4% for the measurement of the un-normalized GSM, 2.4% for the plaque area, and 2.8% for the NORM1 normalized GSM (Table 3). Manual delineation results showed a greater amount of variation (mean coefficients of variation were 7.7% for the un-normalized GSM and 8.0% for the plaque area compared with 5.4% and 4.0%, respectively, for our method), due to the greater subjectivity of the manual delineation process (Table 4). However, the mean difference in GSM measurements between the two techniques was 0.1 grey levels (Figure 8) and did not differ significantly from zero (p = 0.77, t-test). The 95% limits of agreement were −7.9 grey levels to +8.0 grey levels.

**Table 3:** Intra-observer coefficients of variation (standard errors) for the measurement of the inter-frame mean GSM (un-normalized and NORM1 normalized) and mean area, for eight plaque samples

**Plaque sample**	**GSM**	**Area**	**NORM1**
px2	1.7% (0.24)	2.9% (0.37)	2.3% (0.29)
px4	0.5% (0.18)	2.3% (0.11)	4.4% (1.39)
px11	1.6% (0.48)	1.4% (0.24)	4.7% (1.53)
px26	1.1% (0.22)	1.5% (0.26)	1.2% (0.30)
px21	1.4% (0.32)	2.5% (0.25)	2.4% (0.56)
px22	1.5% (0.45)	2.9% (0.29)	1.7% (0.43)
px12	1.8% (0.44)	3.3% (0.22)	2.4% (0.66)
px5	1.4% (0.23)	2.3% (0.23)	3.3% (0.46)
**Column means**	1.4% (0.32)	2.4% (0.24)	2.8% (0.70)

**Table 4:** Comparison with manual delineation for eight selected plaque samples

**Plaque sample**	**Our method**						**Manual delineation**					
	**GSM**			**Area**			**GSM**			**Area**		
	**mean**	**s.d.**	**CV**	**mean (mm**^**2**^**)**	**s.d. (mm**^**2**^**)**	**CV**	**mean**	**s.d.**	**CV**	**mean (mm**^**2**^**)**	**s.d. (mm**^**2**^**)**	**CV**
px2	32.3	2.80	8.7%	27.9	1.12	4.0%	28.7	3.88	13.5%	27.5	2.10	7.6%
px4	76.9	2.22	2.9%	11.3	1.05	9.3%	78.1	4.57	5.9%	11.0	2.03	18.4%
px11	70.2	1.27	1.8%	38.0	0.23	0.61%	73.0	3.36	4.6%	37.4	0.40	1.1%
px26	43.4	1.49	3.4%	39.5	0.94	2.4%	45.9	2.27	4.9%	36.9	1.61	4.4%
px21	52.4	2.35	4.5%	22.7	1.09	4.8%	51.3	2.86	5.6%	22.1	1.80	8.1%
px22	67.4	2.15	3.2%	21.5	0.80	3.7%	67.6	3.54	5.2%	21.3	1.75	8.2%
px12	54.0	3.72	6.9%	14.9	0.39	2.6%	52.9	4.47	8.5%	15.0	1.23	8.2%
px5	36.9	4.31	11.7%	22.0	0.96	4.4%	36.0	4.66	13.0%	22.4	1.71	7.7%
**Column means**	54.2	2.54	5.4%	24.7	0.82	4.0%	54.2	3.70	7.7%	24.2	1.58	8.0%

*GSM* values are un-normalized and *CV* is the coefficient of variation (s.d./mean).

## Discussion

Our investigation highlighted variations in the GSM and area of plaques when measured on a frame-by-frame basis throughout ultrasound image sequences. Image normalisation did not reduce the extent of the GSM variations and in some cases resulted in greater variation. These results demonstrate that frame-by-frame variations in the plaque GSM cannot be offset by applying normalisation factors based on the selection of blood and adventitia regions in one of the frames. Furthermore, selecting separate reference regions in all images introduced an additional source of variability due to the subjective nature of the process. The reference regions were user-selected and not computerized as they are easily identified by the operator and it would have been difficult to ensure the accuracy of a computerized selection. In NORM1 normalisation, the coefficients of variation for GSM changed after normalization for some plaques. This occurred when the blood reference regions had non-zero GSM, causing an intercept to be introduced into the linear relationship between the normalized and un-normalized greyscale values. Furthermore, as GSM values are limited to the range 0 to 255, normalization could result in the clipping of the GSM values outside this range, thus affecting the coefficients of variation. The increased coefficients of variation for GSM, in the case of the NORM2 normalisation, provided evidence that the frame-by-frame variations seen in GSM were unlikely to be, at least significantly, due to a general temporal variability in the overall image brightness.

The extent of the GSM variations seen in the study were similar in magnitude to those reported by Elatrozy et al. [12], who found that, after normalisation, the coefficient of variation among 4 different observers was 4.7% for the GSM of plaques. However, the variations captured by that study did not include variations that may have been observed to the selection of different still images as each of the four observers appeared to have used the same image to assess the GSM. Therefore, the true inter-observer variabilities may have been greater than that suggested by the results of that study.

The findings of our study have two implications. Firstly, in the case of studies which have already considered intra/inter-observer variabilities, the variabilities found may have been under-estimated unless the same image frames were not used in multiple assessments of the GSM. Secondly and conversely, in the case of inter/intra-sonographer or across-study variabilities of GSM measurements, some of these variabilities may have been due to the selection of different image frames corresponding to the differences in the exact cross-sections being imaged.

The variations seen in the plaque GSM and area may be due to a number of different factors. While changes to the acquisition settings during a single acquisition would not be expected, changes in the plaque GSM could occur, for example, if the distance between the plaque and the transducer face changed during an acquisition. Patient or probe motion may also change the location and orientation of the scan plane with respect to the plaque being imaged, affecting both the measured GSM and the observed cross-sectional area. These are likely to be significant contributors to the variations seen in the GSM and area of plaques in this study. Deformation or compression of the plaque under the pulse pressures may also cause changes in the measured plaque GSM and cross-sectional area and the observation of periodical variations with physiologically reasonable frequencies in the plaque GSM and area for several of the plaques provided evidence to support this hypothesis. However, it should be noted that such cyclic variations could also have been caused by periodic variations in the scan plane location and orientation due to out-of-plane plaque, patient, or probe motion. The poor correlation between the inter-frame coefficients of variation of GSM and plaque area suggested that at least some of the variations seen in the plaque GSM were likely to have occurred due to factors other than changes in the observed plaque area. This was also supported by the results of the regression analysis, which did not highlight the parameters based on the plaque area as being statistically significant contributors to the variabilities seen, across plaque samples, in the extent of the inter-frame GSM variations.

Other factors that may have caused apparent changes in the plaque GSM and cross-sectional area included unclear plaque boundaries (e.g. poor quality image or substantial image noise), which may have caused fluctuations in the detected plaque boundaries. However, the images used in this study were of sufficiently good quality that any variations due to such fluctuations were not thought to be a major contributor to the GSM variations observed.

The statistically significant difference found in the mean GSM of plaques in the symptomatic and asymptomatic groups is in accord with previous findings that have shown symptomatic plaques, in general, had lower GSM [3,4]. The differences between the two groups in the case of the parameters describing the inter-frame variations in the GSM were not statistically significant which was plausible as out-of-plane plaque, patient, and probe motion appeared to be a significant sources of variation for GSM measurements.

Manual delineation of the plaque boundaries separately for each image frame was found to increase the extent of the frame-by-frame variations observed in the plaque GSM and area (7.7% and 8.0%, respectively, compared with 5.4% and 4.0% for our method) due to the greater subjectivity of the manual delineation process.

The main limitations of our study were the use of two dimensional ultrasound and the absence of any attempts to fix the scan plane location and orientation with respect to the plaque being imaged, other than those measures normally taken in the clinics (e.g. holding the probe fixed and asking the patient to remain still). It should be remembered that the method of ultrasound acquisition commonly used in carotid plaque GSM studies, namely two dimensional ultrasound, provides only a cross section of the whole plaque volume. Since the plaque GSM measured using two dimensional techniques reflects only a cross-section, these measurements are susceptible to variations due to out-of-plane plaque, probe and patient motion. Studies incorporating three dimensional techniques may overcome this limitation and enable further investigation of the nature of any intrinsic frame-by-frame variations in the plaque GSM. Such follow-up studies may also identify whether any inter-frame variations seen in the GSM and volume of plaques can provide additional insight into the dynamic behaviour of carotid plaques, thus improving clinical utility.

Another limitation of our assessment of the plaque GSM and cross-sectional area was with regard to anechoic regions of plaques and regions of plaques in areas of acoustic shadowing. These regions were excluded from analysis. The cross-sectional areas of plaques that had such regions were, thus, under-estimated and neither the plaque area nor the GSM reflected the true values. The excluded regions also had an effect on the magnitude of the frame-by-frame variations that were measured for the affected plaques. In the case of anechoic regions of plaques, the inclusion of the anechoic regions would have reduced the magnitude of the variations observed. However, this would have been only because of the absence of echogenicity in these regions. In the case of the regions of acoustic shadowing, these regions need to be excluded from analysis due to the absence or deterioration of plaque texture information resulting from acoustic shadowing. Although Colour Doppler is useful for subjectively defining the plaque-lumen boundary, it is not suitable for quantifying the plaque area throughout the cardiac cycle, since colour filling of the lumen is dependent on blood flow velocity [17]. Nevertheless, our results demonstrated that variations in the plaque GSM and cross-sectional area were observed, in the visible parts of the plaques that were not in regions of acoustic shadowing. Such variations are important as they could lead to an error in a potential diagnostic test that uses the GSM as the selection criterion, particularly for plaques of intermediate echogenicity where a coefficient of variation of 5% may provide enough bias to move the plaque between the high-risk and low-risk groups. As the plaque GSM is not generally used to inform clinical decision making, these variations do not currently have a clinical impact. Nevertheless, the variations should be appreciated for research studies which increasingly utilize the plaque GSM.

Our results did not find the cross-sectional plaque area to affect the extent of the frame-by-frame variations observed in the plaque GSM significantly. A major source of variation in the inter-frame plaque GSM may in fact be the movement of the plaque cross-section with respect to the scan plane and this may be a bigger problem for smaller plaques. However, our results showed that the observed GSM could vary on a frame-by-frame basis substantially for large plaques as well the minor stenoses.

Since previous studies typically quantified GSM in single frames of ultrasound images, the variations found in this study have been previously neglected. Improved attempts to standardise GSM measurements and reduce variability between centres should account for these findings, for example, by performing GSM measurements at peak systolic/diastolic frames or by carrying out an assessment of the average GSM throughout the cardiac cycle. Techniques such as GSM assessment using multiple cross-sectional views of plaques and plaque texture analysis can also be used to improve diagnostic precision compared to a single cross-sectional assessment. The best option would be to carry out these processes in three dimensions, however, three dimensional ultrasound techniques are still under development and are not widely available. It should be noted that we do not propose the technique we have used in our study as a replacement for other methods but we highlight the variations in plaque GSM and cross-sectional area that may be observed on a frame-by-frame basis using single-view, two dimensional ultrasound.

## Conclusions

In conclusion, this investigation found that the GSM of carotid artery plaques can vary when measured on a frame-by-frame basis throughout ultrasound image sequences. These variations affect the reproducibility of studies and have implications for the use of GSM as a predictor of cerebrovascular events. Future studies looking at the GSM of carotid artery plaques may need to take these variations into consideration.

## Competing interests

The authors declare that they have no competing interests.

## Authors’ contributions

The study was conceived by KVR and BK. Ultrasound data were collected by TCH. Algorithm development and analyses were carried out by BK. All authors contributed to the interpretation and presentation of the results and, read and approved the final manuscript.

## Supplementary Material

Additional file 1 Plaque tracking sample. Click here for file
